# Target Gene Analysis by Microarrays and Chromatin Immunoprecipitation Identifies HEY Proteins as Highly Redundant bHLH Repressors

**DOI:** 10.1371/journal.pgen.1002728

**Published:** 2012-05-17

**Authors:** Julia Heisig, David Weber, Eva Englberger, Anja Winkler, Susanne Kneitz, Wing-Kin Sung, Elmar Wolf, Martin Eilers, Chia-Lin Wei, Manfred Gessler

**Affiliations:** 1 Developmental Biochemistry, Theodor-Boveri-Institute, Biocenter, University of Wuerzburg, Wuerzburg, Germany; 2 Laboratory for Microarray Applications, and Physiological Chemistry I, Theodor-Boveri-Institute, Biocenter, University of Wuerzburg, Wuerzburg, Germany; 3 Genome Institute of Singapore, Singapore, Singapore; 4 Biochemistry and Molecular Biology, Theodor-Boveri-Institute, Biocenter, University of Wuerzburg, Wuerzburg, Germany; University of Cambridge, United Kingdom

## Abstract

HEY bHLH transcription factors have been shown to regulate multiple key steps in cardiovascular development. They can be induced by activated NOTCH receptors, but other upstream stimuli mediated by TGFß and BMP receptors may elicit a similar response. While the basic and helix-loop-helix domains exhibit strong similarity, large parts of the proteins are still unique and may serve divergent functions. The striking overlap of cardiac defects in *HEY2* and combined *HEY1/HEYL* knockout mice suggested that all three *HEY* genes fulfill overlapping function in target cells. We therefore sought to identify target genes for HEY proteins by microarray expression and ChIPseq analyses in HEK293 cells, cardiomyocytes, and murine hearts. HEY proteins were found to modulate expression of their target gene to a rather limited extent, but with striking functional interchangeability between HEY factors. Chromatin immunoprecipitation revealed a much greater number of potential binding sites that again largely overlap between HEY factors. Binding sites are clustered in the proximal promoter region especially of transcriptional regulators or developmental control genes. Multiple lines of evidence suggest that HEY proteins primarily act as direct transcriptional repressors, while gene activation seems to be due to secondary or indirect effects. Mutagenesis of putative DNA binding residues supports the notion of direct DNA binding. While class B E-box sequences (CACGYG) clearly represent preferred target sequences, there must be additional and more loosely defined modes of DNA binding since many of the target promoters that are efficiently bound by HEY proteins do not contain an E-box motif. These data clearly establish the three HEY bHLH factors as highly redundant transcriptional repressors *in vitro* and *in vivo*, which explains the combinatorial action observed in different tissues with overlapping expression.

## Author Summary

NOTCH signaling is a central developmental pathway that influences a multitude of cell fate decisions and differentiation steps as well as later tissue homeostasis and regeneration. The three *HEY* genes encode basic helix-loop-helix transcription factors that are critical effectors to convey signaling by NOTCH receptors and similar signaling systems. This is underscored by the multitude of developmental defects observed in *HEY* single- and double-mutant mice. The mode of action of HEY proteins remained largely unexplored, however. By gene expression analysis and chromatin immunoprecipitation we have now identified a large set of HEY target genes. While only 500–2,000 mRNAs are regulated by HEY1 or HEY2, there are around 10,000 binding sites in the genome. HEY proteins act as transcriptional repressors that bind close to transcriptional start sites in all cases tested. In contrast, gene activation seems to be mediated by indirect/secondary mechanisms. The extent of regulation is rather limited, implicating *HEY* genes in modulating rather than switching on or off target gene expression. All our *in vitro* and *in vivo* data point to a high degree of redundancy between the three *HEY* genes, suggesting that tissue specific patterns and expression levels determine the final outcome of HEY induced cellular responses.

## Introduction

NOTCH signaling is a key regulatory pathway for cardiovascular development and homeostasis [1]. Its receptors mainly act through transcriptional activation of target genes by a complex of the NOTCH intracellular domain, released by gamma-secretase, the transcription factor CBF1 (RBP-Jk) and the Mastermind co-activator proteins (Maml1-3). Without NOTCH binding CBF1 has a repressive function and associates with additional co-repressor proteins. Upon activation different and in part cell type specific target genes are induced, the most prominent ones encoding members of the HEY and HES family of bHLH repressor proteins. There are three *HEY* genes (*HEY1*, *HEY2* and *HEYL*) and several *HES* genes, with *HES1* being the closest relative. All are related to the Drosophila *hairy* and *Enhancer-of-split* genes, which are well known transcriptional repressor proteins. HEY and HES proteins have a similar domain architecture with a DNA binding and dimer-forming bHLH (basic helix-loop-helix) region, an Orange domain that may also participate in dimerization and conserved C-terminal WRPW (HES) or YRPW (HEY) motifs. The WRPW peptide mediates interactions with groucho-type co-repressor proteins, but YRPW interaction partners for HEY proteins are still unknown.

Mouse knockout studies have revealed a striking overlap in phenotypes between *NOTCH* and *HES* or *HEY* mutants suggesting that these bHLH factors convey a significant fraction of the known biological responses [1], [2]. Loss of *HEY2* or *HEY1*/*HEYL* leads to identical cardiac phenotypes with ventricular septum defects (VSD) and valve anomalies that appear to be due to an impaired EMT process of endocardial cells in the atrioventricular canal [3]. Since *HEY1* and *HEYL* single knockouts do not show evidence of cardiac developmental defects these genes are obviously less critical in this process. Nevertheless, the overlap in endocardial expression and the overlap in phenotypes clearly argue for a combined and partially redundant action of all three *HEY* genes. Interestingly, there is also a cooperation of *HEY1* and *HEYL* in skeletal muscle since double knockout mice lack quiescent satellite cells, which are essential for regeneration [4]. When *HEY1* and *HEY2* are deleted together a much earlier embryonic vascular defect is observed with failure of angiogenic remodeling and a lack of arterial differentiation [5], [6]. Additional critical sites for *HEY* functions are the inner ear [7], brain [8] and bone [9]. The apparent redundancy of HEY factors in many sites and their high degree of sequence identity in the bHLH region suggest that they may be functionally interchangeable, but there are also claims for a completely different function of *HEYL* e.g. in neurogenesis [10]. Evidence for this scenario is limited, however.

Despite extensive analyses of mouse phenotypes surprisingly little is known about the direct targets of *HEY* or *HES* genes [2]. Microarray analyses of overexpressing cells or tissues from knockout animals have provided evidence for *HEY* dependent changes in gene expression in several settings [11]–[14]. There is very little overlap between target lists, however, and evidence for direct regulation of these genes by HEY proteins is largely lacking.

To better characterize the network of genes that mediate NOTCH-HEY signaling effects in target cells we generated HEK293 cells that express HEY1, HEY2 or HEYL in a highly regulatable fashion. These cells were used to search for HEY-dependent changes in transcript levels by microarray analysis and to identify direct binding sites of all three HEY factors in target genes. We could define putative binding motifs and validate DNA targets by promoter analysis. Analysis of cardiac tissue from knockout mice validates a number of these genes as direct in vivo targets.

## Results

### Target gene identification in cells with regulatable HEY genes

To identify target genes of HEY factors we employed HEK293 cells with tightly regulated HEY transgene expression. HEK293 cells were chosen since they express endogenous *HEY* genes at significant levels ensuring that these cells are capable of responding appropriately. According to transcript profiling *HEY1* ranks as number 525 of all expressed genes, while *HEY2* and *HEYL* are expressed at lower levels with a rank of around 10.000 [15]. To generate a system with tunable *HEY* gene expression, cells were first transfected with pWHE134 [16], encoding a reverse tetracycline transactivator (rtTA) plus a tetracycline-dependent repressor (tetR-*KRAB*) driven by a CMV promoter (293tet cells). Flag-HA-(FTH)-tagged *HEY1* and Flag-*HEY2* sequences under the control of a tetracycline-responsive promoter were subsequently introduced via lentiviral vectors (for details see Materials and Methods and Figure S1A–S1D). For some of the experiments Flag-Strep-(FS)-tagged constructs were employed with similar results using transposon-mediated insertion (FS-*HEY1*, FS-*HEY2*, and FS-*HEYL*). The N-terminal epitope tags do not affect localization or transcriptional activity of the proteins in vitro. Western Blot analysis and quantitative RT-PCR of individual clones confirmed tightly regulated doxycycline (Dox)-dependent expression (Figure S1E). Experiments were either conducted with 30–50 ng/ml Dox for low level expression (e.g. raising endogenous *HEY1* mRNA level by 2–10 fold) or with 1–2 µg/ml Dox for stronger overexpression.

**Figure 1:**
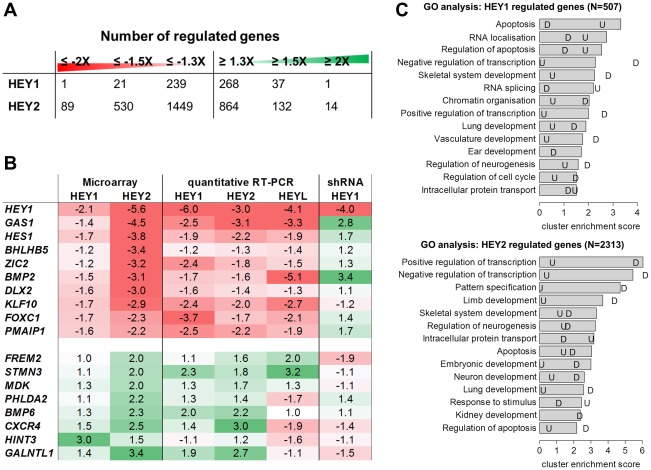
HEY target gene validation and gene ontology analysis. (A) Number of down- and upregulated genes from microarray analysis of HEK293 cells after induction of HEY1 or HEY2. (B) Real-time RT-PCR validation of gene regulation by inducible *HEY1*, *HEY2*, *HEYL* expression and upon *HEY1* knock-down (shRNA). Shown is the fold change between the induced and uninduced state. Note that microarray probes and real-time PCR primers only recognize the endogenous *HEY* transcripts. In the overexpression situation total *HEY1*, *HEY2* and *HEYL* were >220 fold induced compared to endogenous levels. (C) GO term analysis was performed to identify biological processes enriched among HEY1 and HEY2 regulated genes (≥1.3 fold). The top 14 annotation clusters are shown according to their cluster enrichment score [−lg(mean p-value)]. Cluster names are based on enriched GO annotations. “U” indicates the corresponding value for upregulated and “D” for down regulated genes.

HEY regulated genes were identified after strong induction for 48 h (293tet-FTH-*Hey1*) or 72 h (293tet-Flag-*Hey2*), respectively, using Affymetrix microarrays. Under more stringent cut-off values only a small number of genes appeared regulated. With relaxed criteria of ≥1.3× similar numbers of up- and down-regulated genes were identified (Figure 1A and Table S1). The number of target genes and the extent of regulation were greater for HEY2, which may result from differences in protein levels due to longer induction, differential potency of the bHLH protein or cell-intrinsic mechanisms. Comparison of gene lists identified a strong overlap, but in all cases the span of regulation is small.

Validation of microarray results was done by quantitative real-time RT-PCR (qRT-PCR) on a subset of genes (Figure 1B). Repression could generally be confirmed and the extent of regulation tended to be higher, in the range of 2–6-fold. For upregulated genes validation was also successful in most cases, but the extent of regulation was more limited. Importantly, values obtained for HEY1 and HEY2 were more similar now, likely due to the same 72 h induction period. Especially for repressed genes the longer induction time may lead to larger changes since the half-life of target mRNAs becomes less of a problem. Expression of the endogenous *HEY1* as well as *HEY2* and the related *HES1* gene was repressed, pointing to a negative feedback loop for these factors.

These experiments were repeated for HEK293 cells with a regulated expression of Flag-Strep-tagged HEYL and all the genes tested exhibited very similar direction and extent of regulation. Thus, the three HEY factors appear functionally interchangeable, at least in HEK293 cells.

Since HEK293 cells express endogenous *HEY1* this may already lead to a repression at baseline. We therefore tested expression of target genes in a *HEY1* knockdown situation. Using stably expressed shRNA we managed to reduce *HEY1* RNA expression by 75%. Even this rather limited reduction had a clear impact on target genes expression (Figure 1B). Several *HEY*-repressed genes were up-regulated up to 3.4-fold (*BMP2*), while at least some of the genes induced upon *HEY* overexpression tended to be repressed by *HEY1* knock-down, further confirming the validity of our target genes.

### Transcription and development as GO terms

Gene ontology analysis of regulated genes identified a striking overrepresentation of genes related to transcriptional control as well as development and differentiation (Figure 1C). The prevalence of transcriptional control genes suggests that *HEY* genes are positioned higher up in the hierarchy of signaling cascades and modulate other transcription factors rather than effector genes. The terms identified for morphogenetic processes include limb/skeletal development, neurogenesis, organogenesis of branching organs (kidney, lung), cardiac and vascular development, which agrees with the dynamic spatio-temporal *HEY* expression patterns in embryos and implicates *HEY* genes in a broad spectrum of developmental decisions. For *HEY1*, apoptosis-related genes are over-represented especially among upregulated genes. However, most of the strongly enriched GO terms are preferentially found for down-regulated genes, indicating that they form a more focused group. These also tend to exhibit higher ratios of expression changes, which is in agreement with the primarily repressive nature expected for HEY bHLH factors.

### Hey proteins directly bind their target promoters

**Figure 2:**
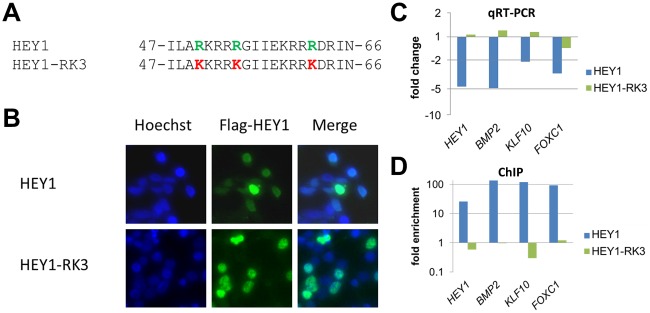
Mutation of putative DNA contacting amino acids in the HEY1 basic domain inactivates its function. (A) Amino acid sequence of the HEY1 basic domain and the HEY1-RK3 mutant with three amino acid exchanges (R50K, R54K, R62K) that likely contact DNA bases. (B) Immunostaining of transfected HEK293T cells with a Flag antibody confirms a preferential nuclear localization of Flag-tagged HEY1 and HEY1-RK3. (C) Real-time RT-PCR shows repression of *HEY1*, *BMP2*, *KLF10* and *FOXC1* by HEY1, but not by HEY1-RK3 (log scale). (D) ChIP analysis confirms enrichment of HEY1 binding sites in target promoters by wild type HEY1, but not by mutant HEY1-RK3 (log scale).

The mode of HEY action has been questioned by publications implicating indirect mechanisms of transcriptional control despite the presence of a classical bHLH domain [summarized in 2]. Especially the lack of E-box target sequences in some of the few known HEY-repressed genes has cast doubt on direct DNA binding as the mode of action. We therefore tested four strong target genes (*HEY1*, *KLF10*, *BMP2* and *FOXC1*) in HEK293 cells for direct HEY binding by chromatin immunoprecipitation (ChIP). 293tet-FTH-*Hey1* cells were induced at low level to avoid oversaturation and HEY-bound DNA was captured using a Flag-antibody. In each case sequences from the proximal promoter region (within 2 kb of the transcriptional start site (TSS)) were efficiently enriched (10- to 60-fold) in Dox-induced cells (see also Figure 2D). Controls with non-induced cells or immunoprecipitations using unspecific IgG antibodies were both negative, demonstrating high specificity. Other conserved sequences further upstream (−1.4 to −6.5 kb from TSS) or intronic regions were not enriched. Experiments with HEY2 and HEYL (not shown) generated essentially the same results, indicating that HEY proteins bind to the proximal promoter regions of target genes in a similar, if not identical fashion.

Further support for direct DNA binding came from experiments with a subtle HEY1 mutant, where conservative point mutations were introduced at three sites in the basic domain, which alter presumptive DNA contacting amino acid residues (R50K, R54K, R62K; HEY1-RK3) (Figure 2). The mutant protein was expressed at similar levels upon Dox induction, it exhibits nuclear localization and it efficiently dimerizes with wild-type HEY1 (not shown). Expression analysis of the target genes *HEY1*, *KLF10*, *BMP2* and *FOXC1* revealed that only wild-type HEY1, but not HEY1-RK3 is capable of repression (Figure 2C). In ChIP analysis HEY1-RK3 did not bind to the corresponding target promoters (Figure 2D). Thus, the basic domain and its presumptive DNA contacting side chains are essential for the transcriptional activity of HEY1.

### ChIPseq analysis for HEY1 and HEY2

Since HEY proteins can directly bind to promoters of target genes we sought to identify the complete repertoire of potential HEY regulated genes through next-generation sequencing of ChIP-enriched DNA fragments (ChIPseq). Non-induced cells were used as a reference. A total of 13–14 million reads were generated for HEY1 and HEY2 and around 90% of these could be mapped back to the human genome (Tables S2, S3, S4). In both cases approximately 10,000 high confidence binding sites could be identified (criteria being a p-value of <10^−5^ and a peak height of ≥10). To validate candidate genes of HEY1 and HEY2, we tested peaks from 23 genes with different height (11 to 380) individually by quantitative PCR (Figure S2). Each binding site could be validated and the same DNA regions were also found to be targets of HEY2 (not shown).

### Characteristics of HEY binding sites

**Figure 3:**
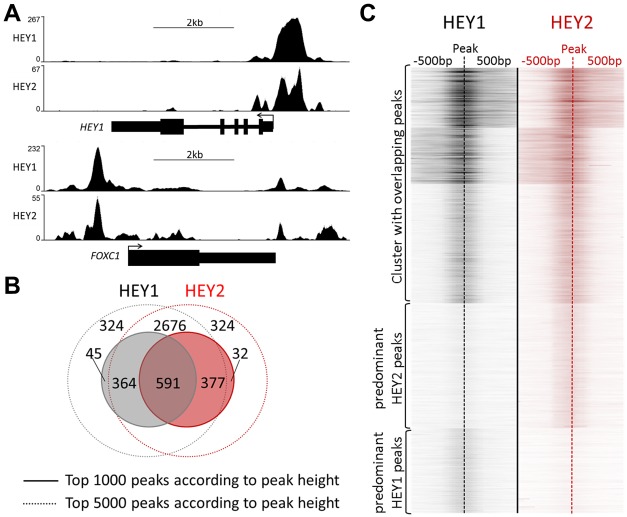
HEY1 and HEY2 have overlapping DNA binding characteristics. (A) ChIPseq profiles for HEY1 and HEY2 are highly similar (examples visualized by the UCSC genome browser). (B) Venn diagram showing the overlap between the top 1000 and top 5000 peaks for HEY1 and HEY2. (C) K-means clustered heat maps of the HEY1 and HEY2 signals at all peak locations (peak summit +/−500 bp), showing three clusters with common peaks and one cluster each with weaker HEY1 versus HEY2 peaks and vice versa.

HEY1 and HEY2 exhibit a remarkable similarity of binding profiles and in most cases peaks of ChIP-enrichment are superimposable (Figure 3A). When binding sites are ranked, 59% of the top 1000 sites are shared between HEY1 and HEY2. A further 37% of these sites are still among the top 5000 binding sites of the other factor, respectively (Figure 3B). Thus, only a small minority of binding sites (≈4%) may be divergent between HEY1 and HEY2 and upon manual inspection most of these are small peaks or the divergence is only of technical nature. The strong similarity of binding is also evident from the heat map generated for all HEY peaks, where very similar distributions of peaks are evident and potential differences seem to be limited to low-scoring sites (Figure 3C).

**Figure 4:**
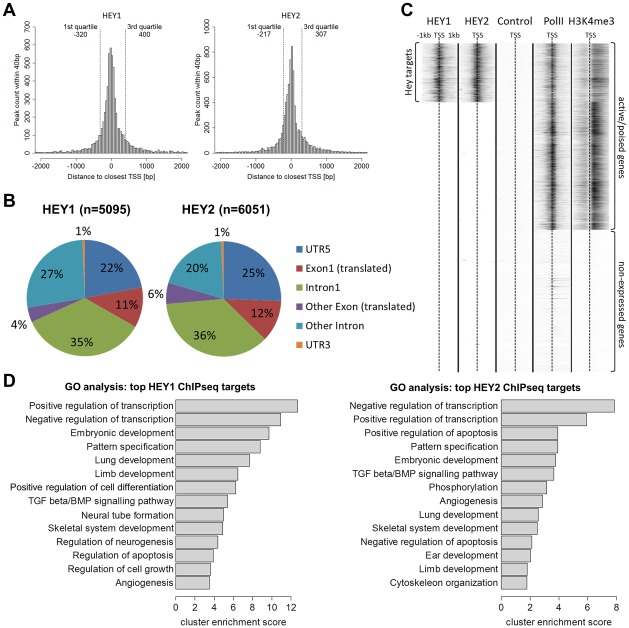
HEY peaks are clustered at transcription start sites of active promoters with a preference for developmental and transcriptional regulatory genes. (A) Histograms show the number of HEY1 and HEY2 peaks within a certain distance to the closest transcription start site (TSS) of the human hg19 refseq genome. Quartiles indicate the window around the median distance containing 50% of all peaks. (B) Pie charts identify main peak locations within genes. (C) K-means clustered heat maps of ChIPseq signals in HEK293 cells for HEY1, HEY2, uninduced control, RNA polymerase II (PolII) and histone-3-lysine-4-trimethylation (H3K4me3). Graph includes all hg19 refseq TSS (+/−1 kb). (D) GO term analysis identifies enriched biological processes among genes with high HEY1 and HEY2 peaks (top 1000 peaks according to height). The top annotation clusters are shown according to their cluster enrichment score [−lg(mean p-value)]. Cluster names are based on enriched GO annotations.

Binding sites are preferentially located in the proximal promoter region of genes or within exon 1: 55–62% of all peaks are within 500 bp of transcriptional start sites (TSS) and 66–76% fall within +/−2 kb (Figure 4A). When the strictly intragenic peaks were counted more than one third each is located in exon 1 or in intron 1, respectively (Figure 4B). This suggests that HEY proteins likely act directly on promoter associated protein complexes and not through long range enhancer or silencer type mechanisms. The vast majority of binding sites (>90%) are located within CpG islands. This is especially true for peaks within +/−2 kb of the TSS (98%) and to a lesser extent for more distal peaks (75%).

HEY binding sites are located preferentially at active or poised promoters exhibiting H3K4me3 histone marks. In HEK293 cells approximately 20.000 promoters are characterized by the presence of Pol II [15] and the histone mark H3K4me3 [17]. Around one third of these sites is also bound by HEY1 or HEY2, representing around 70% of all Hey peaks (Figure 4C). In contrast, there is no evidence at all for HEY binding at silent promoters that lack Pol II/H3K4me3 marks. HEY bound active promoters have somewhat reduced average H3K4me3 values, which may correspond to the repressive capacity of HEY proteins. Gene Ontology analysis of the top 1000 peaks revealed that the promoters bound by HEY proteins are strongly biased towards transcriptional control and embryonic development genes (Figure 4D). This corresponds well to the data obtained from the microarray analyses described above.

### HEYL binds to the same target sites

The striking similarity of HEY1 and HEY2 binding patterns posed the question whether HEYL has the same preferences. This is clearly the case for genes used for ChIPseq validation, listed in Figure S2 (data not shown). Preliminary analysis of ChIPseq data from induced 293tet-FS-HEYL cells revealed that the vast majority of HEY1/2 bound sequences are again bound by HEYL (95% of Hey1/2 peaks, Table S5). We also identified a large number of additional binding sites that tend to be barely present and/or not significant in the analysis of HEY1 and HEY2 (Table S5). HeyL was expressed at somewhat higher levels compared to Hey1/2, which may contribute to the detection of additional, previously not significant peaks. On the other hand, we have little evidence to major changes in Hey binding when cells were induced at lower or higher levels or even transiently transfected. Therefore the basis for the increase in binding sites for HeyL will have to be clarified in future studies before final conclusions can be drawn. Nevertheless, all three HEY proteins appear to bind to the same core of genomic sites with very similar preferences.

### HEY binding motifs

**Figure 5:**
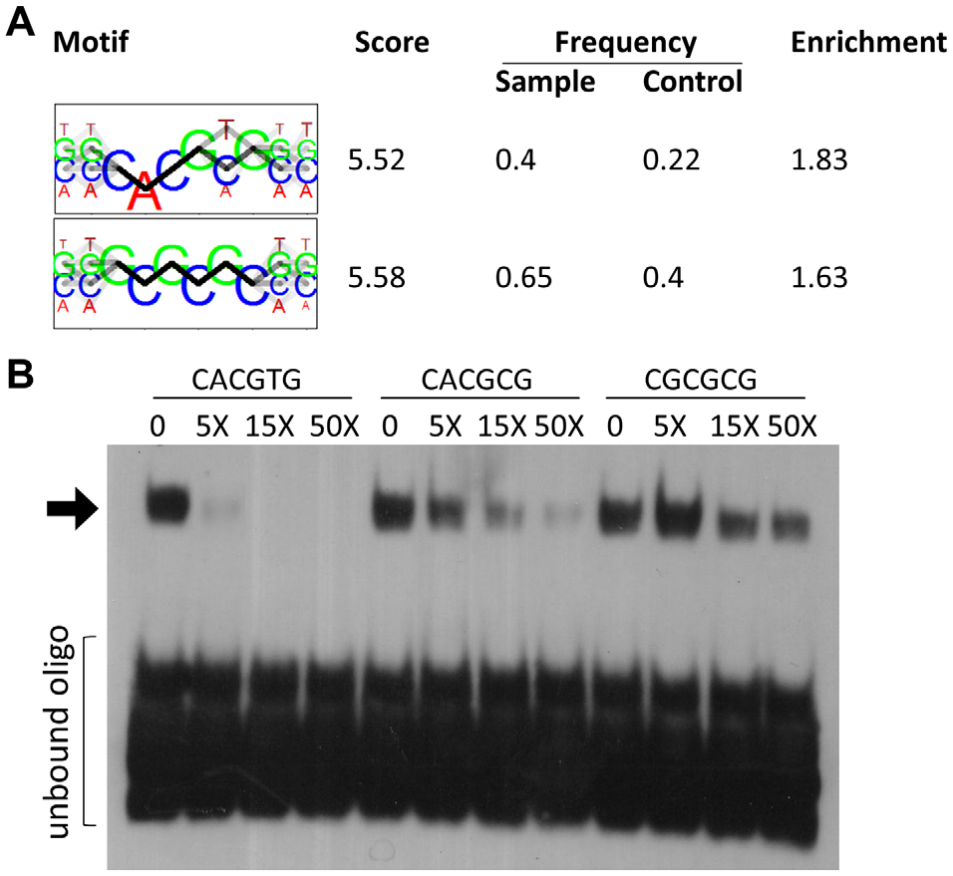
HEY binding motifs identified in silico are bound by HEY proteins in electrophoretic mobility shift assays. (A) The two top motifs for DNA binding by HEY1 identified by de-novo motif discovery using the R package motifRG, based on 200 bp sequences at the summit of the 300 highest HEY1 peaks and 300 matched control sequences with similar GC-content and TSS-distance. (B) Recombinant MBP-HEY1 protein interacts with CACGTG. Binding of the biotin labeled CACGTG probe is competed increasingly by 5-, 15- and 50-fold molar excess of unlabeled CACGTG, CACGCG and CGCGCG-probes. The latter are the least effective. Shifted oligonucleotide indicated by arrow.

To identify potential DNA binding motifs for HEY1 or HEY2 we searched the top 300 target sites (+/−100 bp of peak location) using bioinformatic tools. Sequence motifs that are overrepresented tend to be highly GC-rich since the average GC content in HEY peak regions is around 85%. To reduce the influence of this bias we carefully selected control regions from a set of promoters that are not bound by HEY factors, but display very similar GC profiles. Using the motifRG package we identified two motifs that resemble E-box sequences (Figure 5A). Through binding site selection we had previously identified a class B E-box motif (CACGTG/CACGCG) as a preferred HEY binding site [18], which turned out to be one of the two sequences in our list. One other sequence (GCGCGC) reached a similar score, but its relevance remains unclear.

Electrophoretic mobility shift assays with purified HEY1 protein expressed in E.coli showed strong E-box binding (CACGTG) and efficient competition by the unlabeled oligonucleotide (Figure 5B). The related CACGCG and CGCGCG sequences were much poorer competitors and their own binding to HEY1 could easily be competed by an excess of the prototypic class B site. Nevertheless, only a fraction of Hey peaks contain the CACGYG E-box sequence, suggesting that in vivo binding may employ an even more relaxed consensus or depend on additional interacting proteins.

### Luciferase assays of target genes

**Figure 6:**
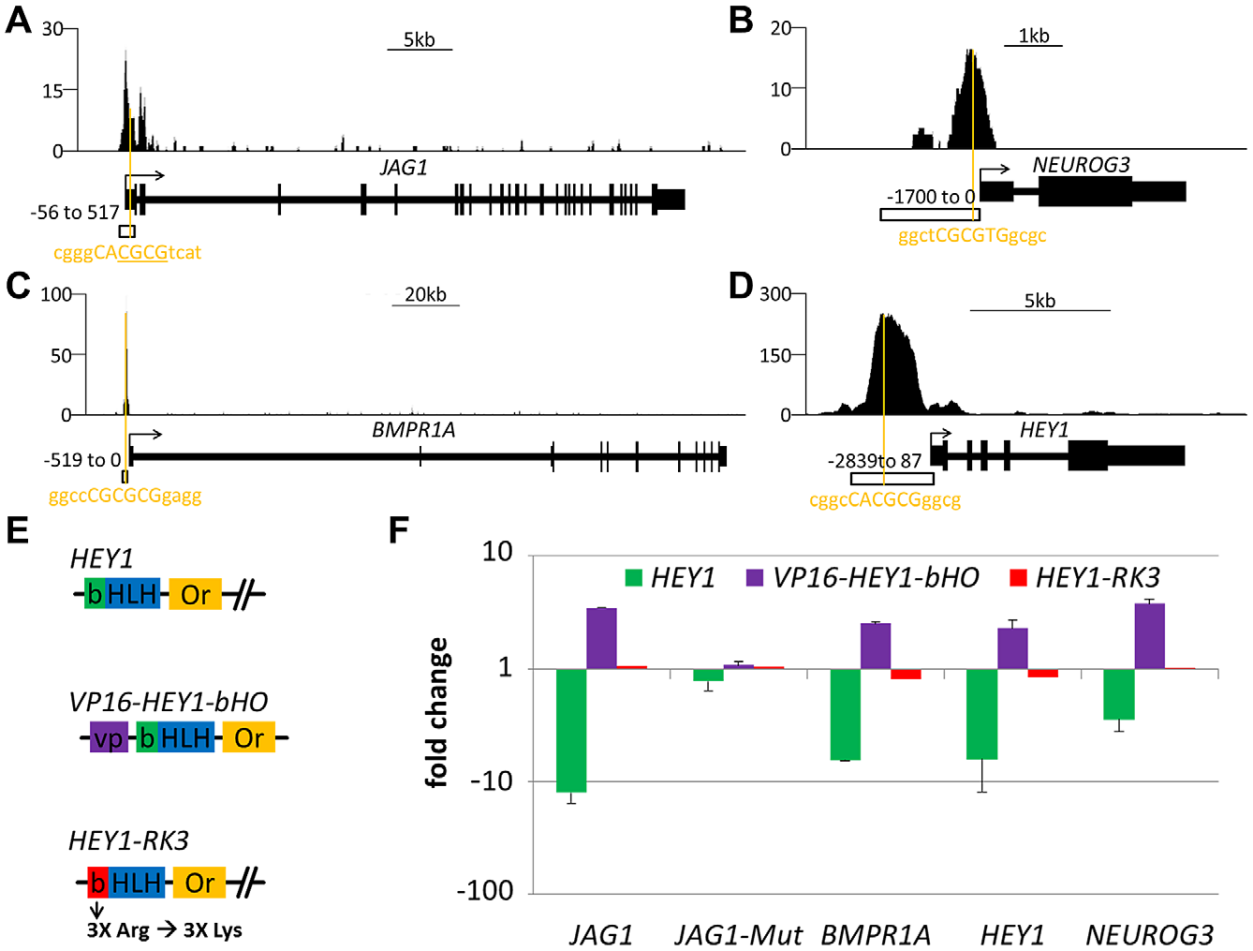
Functional analysis of HEY target promoters. HEY1 ChIPseq signal for the (A) *JAG1* (B) *NEUROG3*, (C) *BMPR1A* and (D) *HEY1* genes. Orange lines indicate the closest potential HEY binding motif under the peak summit. Open boxes indicate the fragment used in the luciferase assay. For *JAG1* a second luciferase construct with a mutated binding site was used (*JAG1-Mut*, deleted nucleotides underlined). (E) Schematic representation of different HEY1 variants used to determine the effect of HEY1 on luciferase-promoter constructs: VP16-HEY1-bHO (HEY1-bHLH-Or inserted after VP16 activator domain), HEY1-RK3 (HEY1 with mutant basic domain). (F) Luciferase reporter analysis in HEK293 cells transiently transfected with reporter-constructs (*JAG1*, *JAG1-Mut*, *BMPR1A*, *HEY1* and *NEUROG3*) and *HEY* expression plasmids or empty vector control (set at 1). Induction/repression was determined from triplicate measurements.

Promoter analysis with luciferase reporter assays validated HEY-dependent repression *in vitro*. In transient co-transfections several promoters like *HEY1*, *JAG1*, *BMPR1A* and *NGN3* were efficiently repressed by cotransfection of *HEY1* (Figure 6). Transfection of an activator construct encoding a fusion of the HEY1 bHLH-Orange sequences with the VP16 activation domain (VP16-HEY1) in turn induced luciferase expression from the same reporter. The HEY1-RK3 protein with its impaired DNA binding capacity was incapable of efficient repression or activation. Each promoter contains at least one sequence motif that could serve as a HEY binding sequence. In the case of *JAG1* a targeted mutation of the putative E-box motif (gggCACGCGtca to gggCAtca) fully abrogated responsiveness to HEY1 or VP16-HEY1. This again demonstrates that HEY proteins directly bind DNA through E-box motifs and mediate repression of their target genes.

### Hey-repressed genes have strong HEY binding sites

**Figure 7:**
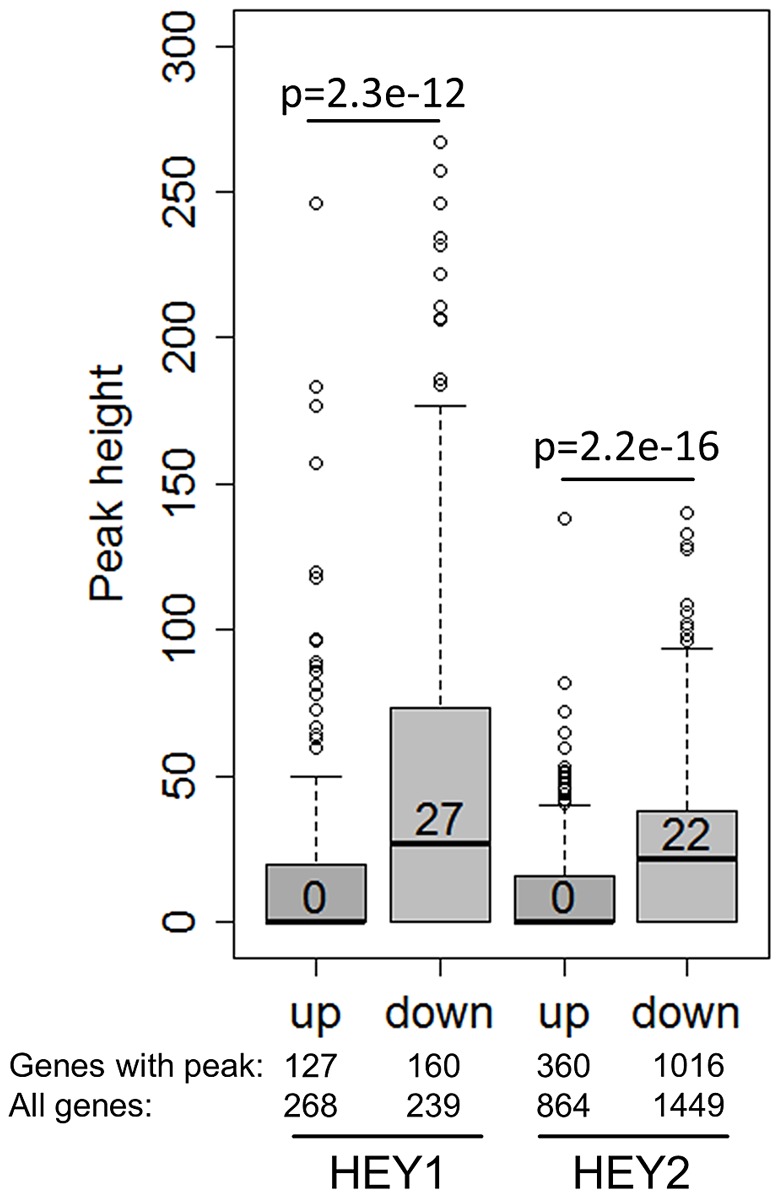
HEY-regulated genes have strong HEY binding sites. Boxplots depict the peak height at HEY1/2 up- and down-regulated genes (≥1.3-fold). On average only down-regulated genes exhibit strong peaks. P-values for a two-sided t-test are given. Shown are also the total number of regulated genes and the number of regulated genes with peaks.

Comparison of target lists for gene regulation and DNA binding further supports the concept of HEY proteins as direct repressors. Especially the genes with stronger repression on mRNA level frequently had ChIP peaks close by and peak height was much higher (median 27 and 22) (Figure 7). Importantly, genes that were induced upon HEY expression did not have significant associated ChIP peaks (median peak height 0). This underscores the notion that repression of transcription appears to be a direct effect of HEY proteins on the corresponding promoters, while gene induction rather tends to be a secondary and indirect phenomenon.

### Validation of HEY target genes in vivo

**Figure 8:**
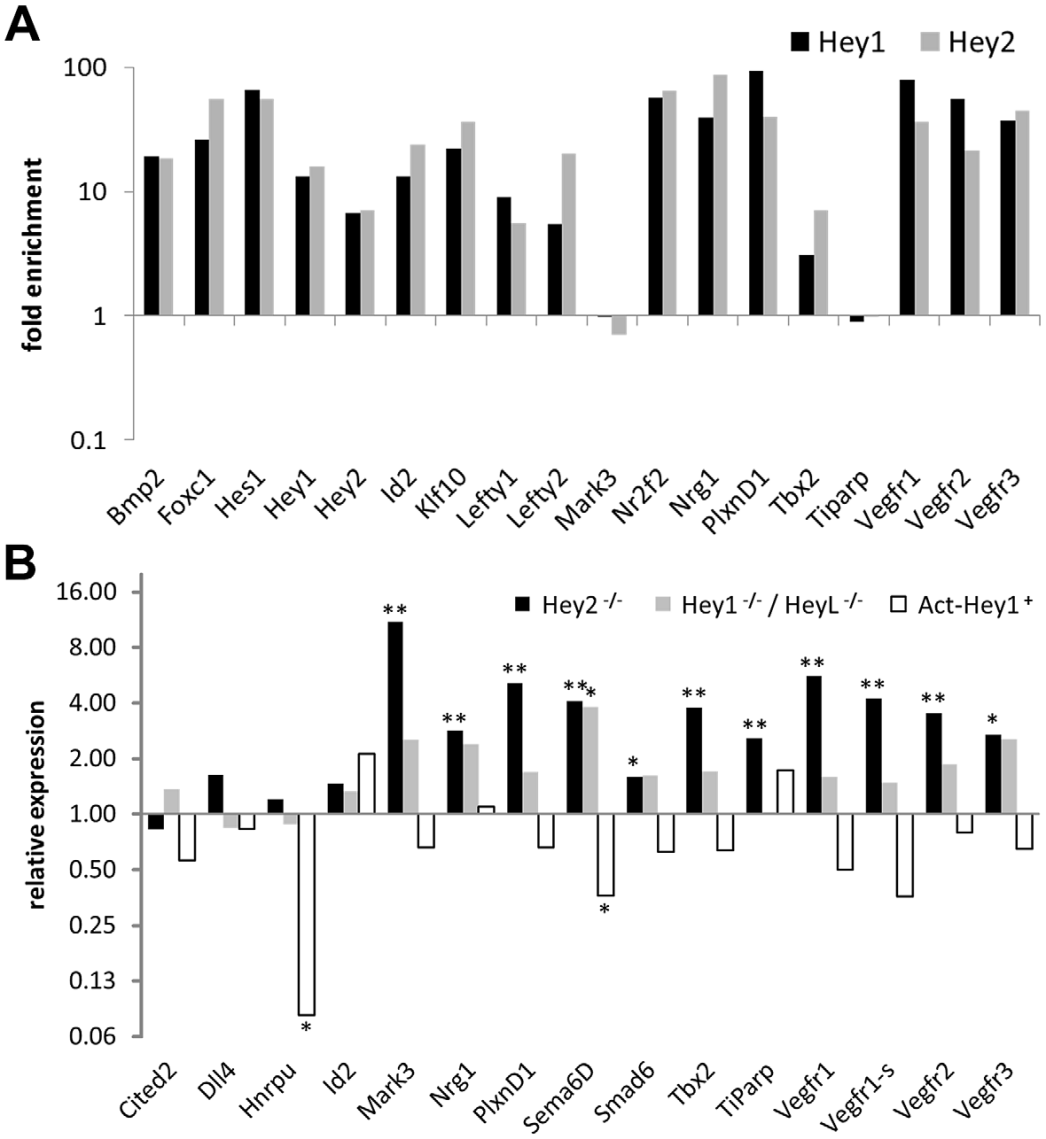
In vivo validation of HEY target genes. (A) Enrichment of Hey1 and Hey2 target sequences from HL-1 cardiomyocytes by ChIP upon transfection with Hey expression vectors compared to EGFP controls. Flanking DNA regions (negative controls) for Hey1, Hey2, Id2 and Nr2f2 were not enriched. (B) Real-time RT-PCR results for cardiac ventricles from *Hey2* ^−/−^, *Hey1* ^−/−^/*HeyL* ^−/−^ and *Hey1* over-expressing (Act-Hey1) E14.5 mouse embryos. Shown is the fold change induction in knockout relative to the wild-type tissue. All genes have HEY-binding peaks identified by ChIPseq. Significant induction is indicated by asterisks (p<0.05 *; p<0.005**).

*Hey* genes are important for development of several organ systems including the heart as reported in numerous knockout studies. We therefore aimed to validate our HEY target genes in the mouse heart. To confirm the presence of HEY binding sites at corresponding genomic locations we repeated our ChIP experiments in HL-1 cells, a murine cardiomyocyte cell line, which maintains cardiac morphology and biochemical and electrophysiological properties in cell culture [19]. We were able to confirm 16 out of 18 HEY binding sites (Figure 8A), indicating that the majority of HEY binding sites detected in HEK293 cells are also present in murine cardiomyocytes.

*Hey2* and *Hey1/L* knockout mice exhibit membranous VSDs and valve defects and overlapping expression in the critical endocardial cells of the AV canal [3], [20]. *Hey2* knockout hearts in addition show evidence of cardiomyopathy in the ventricles, which corresponds well to the fact that ventricular cardiomyocytes express only *Hey2*, but not *Hey1* or *HeyL*. We therefore tested dissected ventricles of *Hey2* ^−/−^ embryos at E14.5 for deregulation of Hey target genes. A series of genes tested exhibited a clear and highly significant up-regulation in knockout embryos by quantitative real-time RT-PCR (Figure 8B). In contrast, *Hey1* and *HeyL* are not expressed in the ventricles and in the knockout situation there is only limited deregulation of the same set of genes where only induction of Sema6d reaches statistical significance. To extend these findings we also tested ventricles from animals with a global Hey1 over-expression [9]. In this case, most of the genes up-regulated in Hey2^−/−^ mice were down-regulated (Figure 8B) with the lower amplitude likely being due to endogenous *Hey2* already being present. This clearly documents that Hey repression of target genes is functional in the mouse *in vivo* with an induction of these genes in the knockout situation.

## Discussion

The strong phenotypes of Hey knockout mice raised the question of potential target genes that may mediate the effects observed in various cell types. To gain such insight we have characterized the genome-wide regulatory potential of HEY proteins by performing microarray gene expression analysis combined with ChIPseq to identify all potential HEY binding sites.

### HEY factors only modulate gene expression

*HEY* genes have been described as repressors of a small number of individually tested target genes that had been identified serendipitously by various means [summarized in 2]. To search for additional HEY regulated genes we chose HEK293 cells as these are easy to manipulate and they express endogenous *HEY* genes suggesting that they can react to altered HEY protein levels in a physiologically relevant manner. We obtained very similar patterns of expression changes for *HEY1* and *HEY2*, both on microarrays as well as in confirmatory real-time RT-PCR. Even the more divergent family member *HEYL* led to concordant regulation of the target genes tested. Surprisingly, the level of regulation was rather limited in all cases. *HEY1* itself is the strongest down-regulated gene (3–6-fold), indicative of an important negative feedback loop. For *HEY2* and *HEYL* the repression was also seen, but less pronounced. This negative feedback loop may be similar to the ones described for *Hes1* and *Hes7* that are important in somitogenesis and neural stem cell biology [21]. The generally modest expression changes suggest that *HEY* genes rather modulate existing gene transcription instead of completely switching expression states. On the other hand, preexisting *HEY1* mRNA and protein in HEK293 cells may already induce a level of repression that can be further enhanced to a limited extent only and this is supported by our experiments with Hey1 shRNA, where an induction of several target genes could be seen. The study by Xin et al. [13] likewise reported a small number of *HEY2* regulated genes, where only three structural genes showed regulation in the range of 5–9-fold, which is in line with our data.

### HEY factors redundantly bind to many target sites

Gene regulation on mRNA level could be due to direct or indirect effects of HEY proteins on target promoters. This distinction became more relevant as HEY proteins led to induction and repression of comparable numbers of transcripts. ChIP analyses are an excellent tool to generate additional evidence for a direct mode of action. In these experiments we relied on a rather limited overexpression of *HEY* genes in order to still mimic a physiological situation. Nevertheless, we identified a very large number of around 10,000 target sites in HEK293 cells with almost identical profiles for HEY1 and HEY2. Differences are mostly restricted to less enriched target sites. This translates to a Pearson's correlation of r = 0.75 between HEY1 and HEY2, which is close to the value of r = 0.83 obtained for biological replicas in other studies [22], indicating that HEY1 and HEY2 regulate the same targets. For HEYL an even larger number of peaks was identified. While the reason for the increased number of binding sites is still unclear, the vast majority of HEY1 and HEY2 peaks were again seen in our HEYL dataset, supporting the idea of strongly overlapping functions.

There is a striking discrepancy, however, between the large number of ChIP peaks and the much smaller number of genes regulated by HEY proteins. The vast majority of binding sites observed may thus not contribute to gene regulation, or else endogenous HEY proteins may have already exhausted the regulatory potential at some of these sites. On the other hand, an overabundance of bound DNA sequences has been observed for other transcription factors before, like the bHLH factors MYC or MYOD that yielded comparable results [23], [24]. Given the probably limited protein concentration it even appears questionable if all sites will be occupied simultaneously in any given cell and rather points to a high turnover rate. For c-MYC, another E-box binding protein, a two-step model of initial binding to open chromatin followed by more relevant sequence specific binding has been put forward [25]. Functionally active binding sites may also emerge only through additional modifications or concomitant binding of additional factors to form fully functional complexes. A possible scenario to explain HEY functions might therefore include a general preference of HEY factors for genes with an open chromatin configuration, where the actual transcriptional change then depends on circumstances like cell type and differentiation status. It remains to be established if and how HEY functions can be described by such models.

### HEY proteins act as direct repressors

The mode of regulation by HEY proteins appears to be rather uniform. The vast majority of binding sites were found in close proximity to transcriptional start sites. This rather implicates HEY proteins in direct interactions with the basal transcriptional machinery or local chromatin at promoters as opposed to long range enhancer type mechanisms. The majority of HEY-repressed genes appears to be direct targets since they contain strong HEY binding sites within the promoter or 5′ UTR regions. On the other hand, genes activated by HEY proteins are likely regulated in an indirect manner: more than half of them do not contain relevant peaks at all and peak height was generally rather small, suggestive of HEY expression leading to a reduction in other critical transcriptional activators for those genes.

Direct repression of target promoters could also be verified in vitro by luciferase reporter assays. As reported in earlier studies by us and others, HEY1/2/L can repress target promoters up to ten-fold [14], [26]. Our current experiments provide important additional evidence for a HEY function as direct transcriptional repressor: Firstly, the bHLH-Orange domain can be turned into an activator of transcription when fused to the strong VP16 activation domain. Furthermore, changing only three arginine residues that presumably contact DNA into lysine completely abolished DNA binding and transcriptional response of this mutant. This clearly establishes HEY proteins as direct DNA binding transcriptional repressors, while gene activation by HEY proteins appears to be indirect as the promoters are largely devoid of HEY target sites.

### HEY proteins target E-box sites

A putative DNA binding motif for HEY proteins of tggCACGYGcca has previously been defined by in vitro oligonucleotide selection [18]. However, in most studies the core consensus E-box site CACGYG was either not present in the small number of putative target promoters analyzed previously, or deletion of related E-box sites did not alter expression of luciferase reporter constructs [summarized in 2], leading to proposals of indirect HEY functions. Here we could show that deletion of an E-box site in the *JAG1* promoter abolishes HEY regulation in luciferase assays. Related findings have recently been published for the *IDE* promoter [27]. This shows that at least for some HEY target genes the E-box motif is required for Hey regulation. De novo motif discovery in our ChIPseq data set also led to the identification of an E-box motif of CACGYG as one of the two top candidates. Finally, a search of all known DNA binding motifs likewise recovered myc-type E-box sequences as being highly enriched (not shown). While these data clearly demonstrated that E-box sequences can be bound by HEY proteins in vivo, this does not fully explain the genomic binding patterns observed since many of the bound regions do not contain such motifs. HEY proteins may either use less stringent criteria for DNA binding in vivo or they might also bind in a sequential manner that initially does not fully rely on sequence specificity as suggested by Perna et al. [25]. Another possibility would be the need for additional cooperating factors that bind in the vicinity or form ternary complexes to ultimately affect gene expression, but this will depend on the characterization of novel HEY binding partners. The observed co-occurrence of binding sites for factors like SP1, E2F, AP2, NRF and EGR is expected at promoter-proximal regions, but may also hint at potential interactions of HEY proteins with some of these factors.

### HEY factors only bind on active and poised promoters

The rather limited extent of gene repression by HEY proteins is also reflected in the chromatin signature of the corresponding promoters. There is a striking overlap of HEY bound sequences with the presence of polymerase II (Pol II) and the active chromatin mark H3K4me3, which are preferentially found at active and poised promoters. This again argues in favor of a modulatory role of HEY proteins with just limited alterations in gene expression. In human ES cells Pol II and H3K4me3 marks have been identified at silent genes as well, however, and it has been suggested that the critical step lies in transcription elongation. Interestingly many developmental regulators fall within this group of genes [28], [29], to which a significant fraction of HEY target genes belongs as well. The location of a large number of HEY binding sites just downstream of the transcriptional start site would ideally position HEY to influence the pausing vs. elongation switch of Pol II.

### Paralogous HEY genes are highly redundant

Previous studies have suggested redundancies between *HEY1*, *HEY2* and *HEYL* that manifest in distinct phenotypes in single and combined KO mice due to partly overlapping expression profiles [3], [5], [6]. The striking overlap in gene regulation and the highly related patterns of ChIPseq peaks indicates that all three HEY proteins indeed elicit very similar responses in a given cell type. This is consistent with the idea that the expression patterns of HEY factors largely define the outcome of knockout studies, whereby no individual, intrinsic functional properties, but overall and cumulative Hey expression levels would be critical. On the other hand, the substantial divergence in the poorly conserved C-terminal half of the proteins is suggestive of a significant potential for paralog-specific functions that may yet have to be uncovered. The identification of either fully shared or paralog-specific protein interaction partners of HEY factors may help to shed light on this important issue.

## Materials and Methods

### Cell culture and generation of inducible cell lines

HEK293 cells were cultured in DMEM medium (PAN Biotech, Aidenbach, Germany) containing 10% FCS, 50 U Penicillin and 50 µg/ml Streptomycin. 293tet cells were generated by transfection with PvuII linearized pWHE134 plasmid [16] using polyethylenimine (3 µg DNA, 6 µl PEI per 6-well plate for 8 h) followed by selection with 0.5 mg/ml G418. HEY expressing cells were produced by lentiviral transduction of 293tet cells with p199-FTH-*hHey1*-iEP, p199-Flag-*mHey2*-iEP constructs based on p199 plasmids [30] (for maps see Figure S1). For regulated HEYL expression pTol2-FS-*mHeyL*-iEins-WHE carrying insulator sequences (HS4ins) and the complete tet-regulatory module from pWHE459 [31] was introduced into HEK293 cells by Tol2-mediated transposition with pKate-N/Tol2 [32] followed by puromycin selection (1 µg/ml). The *HEY1* knockdown was generated by lentiviral transduction of HEK293 cell with shRNA vectors (Open Biosystems clone ID V3LHS_404238). In all cases individual colonies were picked and validated separately. The HEY1-RK3 mutant was generated by PCR-mediated mutagenesis using primers spanning the altered sites. All constructs were verified by sequencing.

HL-1 cells were cultured in Claycomb medium (Sigma-Aldrich, Munich, Germany) containing 10% FCS, 100 µM norepinephrine, 4 mM L-glutamine, 50 U Penicillin and 50 µg/ml Streptomycin. For ChIP 5*10^6^ HL-1 cells were transiently transfected with 8 µg plasmid DNA using 100 µl Ingenio Buffer (Mirrus, Madison, USA) and the Amaxa Nucleofector II electroporator (program T-20, Lonza, Basel, Switzerland). After 48 h of culture, cells were used for ChIP.

### Mouse lines

Hey1, Hey2 and HeyL knockout lines have been described before [3], [5]. The Act-Hey1 transgenic line expressing Hey1 under the control of the ß-actin promoter was obtained from M. Susa (NIBR, Basel) [9].

### RNA isolation

Total RNA was extracted either from cells or tissue samples using TriFast (peqGOLD, Peqlab, Germany, Erlangen) according to the manufacturer's protocol and quantified by OD_260 nm_ measurements using a spectrophotometer (NanoDrop ND 1000, Peqlab).

### Microarray analysis

Total RNA of control and dox-induced cells (1–2 µg/ml doxycycline for 48–72 h) was used for microarray analysis on Human Genome U133 Plus 2.0 Gene Arrays (Affymetrix, Santa Clara, CA). Labeling and washing were performed according to the standard Affymetrix protocol. The arrays were scanned using a GeneChip Scanner 3000 (Affymetrix). Data analysis and quality control was done using different R packages from the Bioconductor project (www.bioconductor.org). Probe sets were summarized using the RMA algorithm and resulting signal intensities were normalized by variance stabilization normalization [33].

### Quantitative real-time RT–PCR (qRT–PCR)

2 µg RNA were reverse transcribed using the Revert Aid First-Strand cDNA synthesis Kit (Fermentas, Lithuania, Vilnius) with oligo(dT) primers. qRT-PCR was performed with an iCycler iQ5™ Real-Time PCR Detection System (BioRad, USA, Hercules). Primer sequences are listed in Table S6. Reactions contained 1/50 of the cDNA reaction and PCR was performed with annealing at 60°C and SybrGreen quantification. PCR products were confirmed by melting curve analysis and agarose gel electrophoresis. The housekeeping gene HPRT was used to normalize expression levels. All measurements were performed at least twice and mean values were calculated.

### ChIP analysis

5*10^6^ HEK293 cells were induced with 50 ng/ml doxycycline for 48 h to obtain low level overexpression of HEY proteins. The same amount of cells was kept uninduced as control. HL-1 cells were transiently transfected with pCS2p-Flag-Hey1, pCS2p-Flag-Hey2 [14] and pll3.7, which was used as control. Cells were harvested as described earlier [34]. Briefly, the cells were fixed with 1% paraformaldehyde for 10 min at room temperature. Fixation was stopped by adding glycine to 0.2 M and cells were washed three times with ice-cold PBS and harvested. All subsequent steps were done at 4°C. The cells were lysed in cell lysis buffer (50 mM Hepes-KOH pH 7.5, 150 mM NaCl, 1 mM EDTA, 1% TritonX-100, 0.1% Deoxycholate, 0.1% SDS) and spun down. The resulting pellet of nuclei was lysed in nuclei lysis buffer (cell lysis buffer containing 1% SDS) and sonicated using a Digital Sonifier W-250 D (Branson Ultrasonics, USA, Danbury). Debris was removed by centrifugation. For immunoprecipitation, Chromatin was diluted five-fold with ChIP buffer (0.01% SDS, 1.1% TritonX-100, 1.1 mM EDTA, 20 mM Tris pH 8.0, 167 mM NaCl) and 550 µl of diluted chromatin was mixed with 45 µl 1∶1 protein G agarose slurry (in cell lysis buffer) and 4 µg antibody (αFlag-M2 or rabbit IgG, Sigma-Aldrich) and incubated overnight. Then, the agarose beads were washed three times with cell lysis buffer, once with washing buffer (50 mM Hepes-KOH pH 7.5, 350 mM NaCl, 1 mM EDTA, 1% TritonX-100, 0.1% Deoxycholat, 0.1% SDS) and once with LiCl washing buffer (10 mM Tris-HCl pH 8.0, 250 mM LiCl, 1 mM EDTA, 0.5% Nonidet P-40, 0.5% SDS). Elution was performed with elution buffer (50 mM Tris pH 8.0, 10 mM EDTA, 1% SDS) at 68°C for 30 minutes. The eluted chromatin was incubated with 0.8 mg/ml Proteinase K and PFA fixation was reversed by incubation at 68°C overnight. The DNA was then purified using phenol-chloroform extraction, precipitated and quantified using PicoGreen (Invitrogen, USA, San Diego).

### ChIPseq

For ChIPseq the same protocol as for ChIP was used in principle, but 2.5*10^8^ cells were employed and after lysis of nuclei, chromatin was spun down at 20.000 rpm (SW 41 TI rotor, Beckman, USA), washed twice with cell lysis buffer and sonicated in 1 ml cell lysis buffer per 100 µl chromatin pellet. For ChIP 10 µg antibody was used. 7–12 ng of ChIP DNA was subjected to sample preparation using the NEBNext ChIPseq sample preparation kit (New England Biolabs, Ipswich, USA) according to the manufacturer's instruction. Briefly, DNA was end-polished with T4 DNA polymerase and kinase. After column purification, Illumina adaptors were ligated to the ChIP DNA fragments. For HEY1 and HEY2 fragments were subjected to 15 cycles of PCR amplification and DNA with a length of 200–350 bp was excised from an agarose gel using Qiagen gel extraction kit. For HEYL 175–225 bp fragments were first excised and then amplified by 18 cycles of PCR. The DNA fragments were sequenced on an Illumina GAIIx platform (Illumina, USA, San Diego). 36 bp sequences were generated and mapped to the hg19 genome by bowtie 0.12.7 [35] with standard parameters. These raw sequencing data were further analyzed using the peak finding algorithm MACS 1.4.1 [36] using sequences from uninduced cells as control to identify the putative binding sites. All peaks with a minimum p-value of 10^−5^ and a minimum height of 10 were included. The uniquely mapping locations for each factor were used to generate the genome-wide intensity profiles, which were visualized using the USCS genome browser. PeakAnalyzer [37] was used to annotate peaks and to calculate overlaps between different bed files. Heat maps were generated using seqMiner 1.2.1 [38] with K-means raw clustering.

### GO term analysis

GO terms analysis was performed with DAVID 6.7 [39] using the functional annotation clustering method and allowing only biological processes. Clusters were named based on interpretation of enriched GO annotations.

### De novo motif discovery

The R-package motifRG (Bioconductor package motifRG, Zizhen Yao, manuscript in preparation) was used to identify binding motifs, using sequences +/−100 bp around the summit of the top 300 highest ranking peaks. Unrelated sequences with a similar distance towards transcription start sites of genes lacking ChIPseq peaks and with similar GC distribution were selected and used as control/background.

### Mutation of JAG1 luciferase construct

The *JAG1* luciferase construct containing the potential Hey binding sequence tgaCGCGTGccc was mutated by cutting with MluI (ACGCGT, Fermentas), followed by Mung Bean Nuclease (Fermentas) treatment and religation using T4 DNA ligase (Fermentas). This results in a four base pair deletion.

### Luciferase assay

For luciferase assays approximately 10^4^ HEK293 cells were transiently transfected with 250 ng of the luciferase promoter construct and 50 ng of the regulatory *HEY* construct in a 24-well format. Cells were harvested after 48 h and lysed in 150 µl lysis buffer (25 mM Glycyl-Glycine pH 7.8, 15 mM MgSO_4_, 15 mM KP_i_, 4 mM EGTA, 1 mM DTT, 1% Trition-X100). After incubating for 10 min at room temperature cells were pelleted and 50 µl of the supernatant were measured in a GLOMAX 96 microplate luminometer (Promega, USA, Madison) using 150 µl assay buffer (lysis buffer with, 1 mM ATP, 0.1 µg/µl D-Luciferin). All measurements were done in triplicates.

### EMSA

EMSA was performed using binding buffer (20 mM Tris pH 7.6, 100 mM KCl, 0.5 mM EDTA, 0.1% Nonidet P-40, 1 mM MgCl_2_, 1 mM DTT, 10% glycerol) and 1 ng recombinant MBP-HEY1 protein, 1 µg poly-dAdT, 5 ng biotin-labeled probe and either 0, 25, 75 or 250 ng competing unlabeled probe in a total volume of 10 µl. After incubating on ice for 30 min, samples were loaded on a 6% polyacrylamide gel and later blotted onto Amersham Hybond N+ membranes (Amersham, UK). Detection was done using the PIERCE LightShift Chemiluminescent EMSA kit according to the manufacturer's recommendations (PIERCE, USA, Rockford).

### Immunofluorescence

HEK293T cells were transfected with either HEY1 or HEY1-RK3 expression plasmids 24 hours prior to fixation with 4% PFA. After blocking with 5% goat serum/0.3% Triton X-100/PBS, the Flag antibody (rabbit; Cell Signaling, Danvers, Massachusetts) was added (1∶800; o/n, 4°C). After washing in PBS at RT the secondary antibody Alexa488-rabbit (Bio-Rad) was used (1∶2000; 1 h, room temperature). Nuclei were stained using Hoechst33342 (1∶10000; Roth, Karlsruhe, Germany) and subsequently cover slides were mounted in Mowiol. Pictures were taken using a Leica AF6000 fluorescence microscope.

### Usage of public data

The ENCODE ChIPseq data for H3k4me3 was downloaded from “ftp://encodeftp.cse.ucsc.edu/pipeline/hg19/wgEncodeUwHistone/” (Producer: University of Washington) [17] and the ChIPseq data for PolII was downloaded from “http://www.ncbi.nlm.nih.gov/gds?term=GSE11892” [15].

## Supporting Information

Figure S1 *HEY* expression vectors and expression controls. Maps of vector constructs used to create stable *HEY* expressing cell lines. For *HEY1* (A) and *HEY2* (B) lentiviral vectors containing Flag-Strep (FS) tagged *HEY1* or *HEY2*, respectively, under control of a tetracycline responsive promoter (TRE-tight) were used. (C) For *HEYL* a vector for transposon mediated insertion was used containing Flag-Strep-tagged *HEYL* under control of a tetracycline responsive promoter. (D) For *HEY1* a lentiviral vector containing Flag-TEV-HA tagged *HEY1* was used in some experiments with identical results. (E) HEY protein expression was verified by Western Blot and real-time RT-PCR of stable cell lines. Cells were harvested after induction with different doxycycline concentrations for 48 h using standard SDS lysis buffers. Western blots on nitrocellulose membranes were developed using the Flag-M2 antibody (Sigma-Aldrich) and anti-mouse-POD as a secondary antibody (Chemicon, Millipore, Billerica, MA, USA) with chemiluminescent detection. The fold induction compared to endogenous *HEY* mRNA levels is shown for the concentrations used for ChIP and RT-PCR experiments (the primers used here amplify endogenous as well as transgene derived *HEY* transcripts). The high induction seen for *HEY2* and *HEYL* is due to the rather low endogenous expression.
(TIF)

Figure S2 Validation of HEY1 ChIPseq data by quantitative PCR. Shown is the fold enrichment of potential HEY1 binding sites from promoter regions identified by ChIPseq compared to non-induced control cells. Genes are ordered according to peak height (from ChIPseq data) as indicated below. CCNB1 was used as a negative control.
(TIF)

Table S1 Gene expression array data for HEY1 and HEY2. Regulated genes (≥1.3×) from microarray experiments of HEK293 cells with doxycycline inducible *HEY1* (1 µg/ml dox for 48 h) and *HEY2* (1 µg/ml dox for 72 h) expression. Genes are sorted according to induced/uninduced ratio.
(XLSX)

Table S2 Statistics of ChIPseq analysis. ChIPseq data for HEK293 with induced (+dox) and uninduced (-dox) HEY expression were generated using the Illumina sequencing platform. Reads were mapped against the human hg19 refseq genome using bowtie with standard parameters. Peaks were identified using MACS. The HEY2 uninduced control was also used as control for HEY1.
(XLSX)

Table S3 ChIPseq peaks for HEY1. Shown are the locations of all HEY1 ChIPseq peaks, by giving the start and the end point of each peak as well as the summit position (location with highest enrichment). tags = total number of reads attributed to peak; pvalue = given as −10*log_10_(pvalue); fold = fold enrichment; FDR = false discovery rate; height = peak height; TSS Distance = distance to closest transcription start site; given are also the location and orientation of the Gene with the closest TSS.
(XLSX)

Table S4 ChIPseq peaks for HEY2. Shown are the locations of all HEY2 ChIPseq peaks, by giving the start and the end point of each peak as well as the summit position (location with highest enrichment). tags = total number of reads attributed to peak; pvalue = given as −10*log_10_(pvalue); fold = fold enrichment; FDR = false discovery rate; height = peak height; TSS Distance = distance to closest transcription start site; given are also the location and orientation of the Gene with the closest TSS.
(XLSX)

Table S5 ChIPseq peaks for HEYL. Shown are the locations of all HEYL ChIPseq peaks, by giving the start and the end point of each peak as well as the summit position (location with highest enrichment). tags = total number of reads attributed to peak; pvalue = given as −10*log_10_(pvalue); fold = fold enrichment; FDR = false discovery rate; height = peak height; TSS Distance = distance to closest transcription start site; given are also the location and orientation of the Gene with the closest TSS.
(XLSX)

Table S6 Sequences of all primers used.
(XLSX)
