# Supplementary figures and images for: Target Gene Analysis by Microarrays and Chromatin Immunoprecipitation Identifies HEY Proteins as Highly Redundant bHLH Repressors
Source: PLoS Genet. 2012 May 17;8(5):e1002728. doi: 10.1371/journal.pgen.1002728 (PMC3355086; doi:10.1371/journal.pgen.1002728)

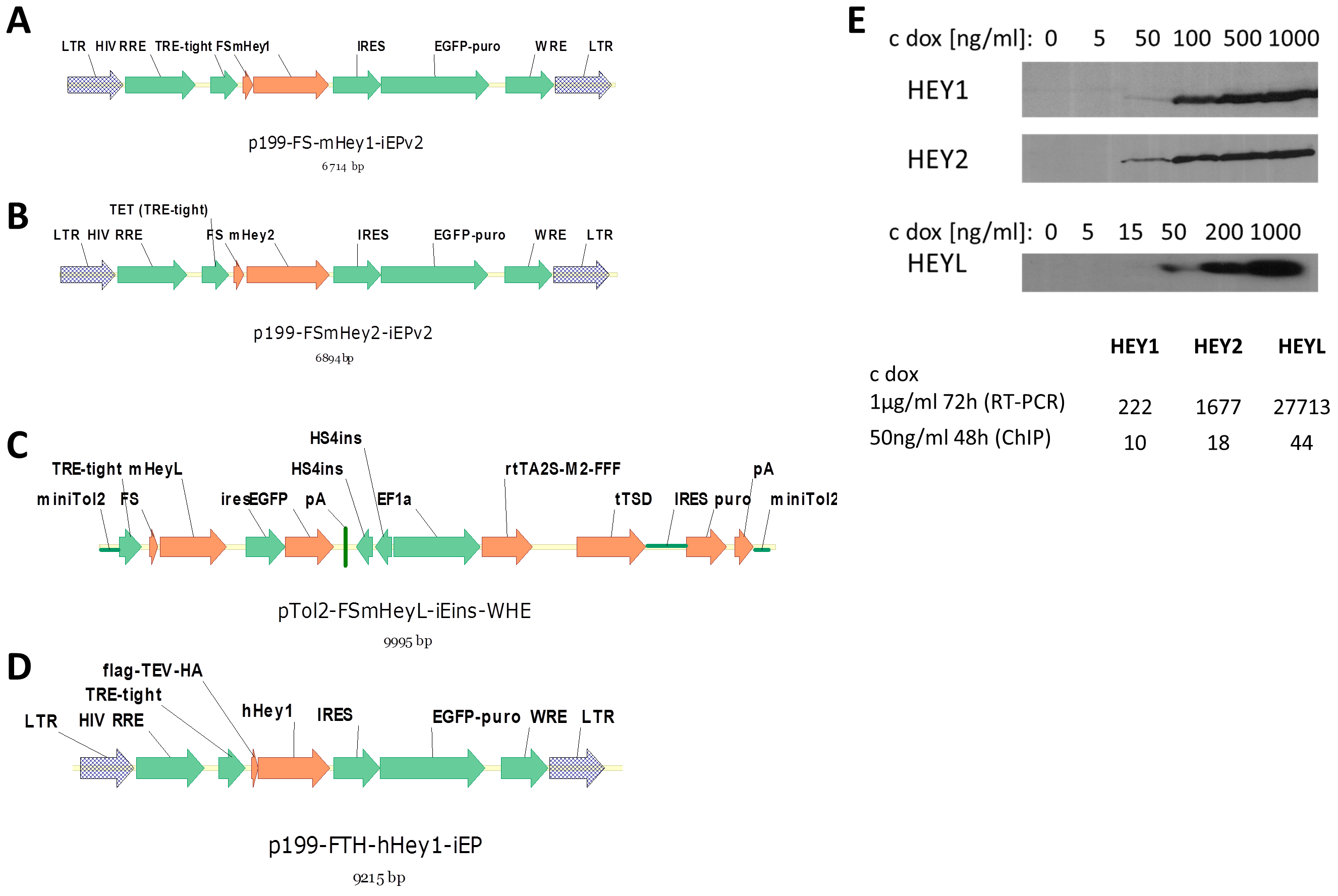

Supplement: Figure S1 — HEY expression vectors and expression controls. Maps of vector constructs used to create stable HEY expressing cell lines. For HEY1 (A) and HEY2 (B) lentiviral vectors containing Flag-Strep (FS) tagged HEY1 or HEY2, respectively, under control of a tetracycline responsive promoter (TRE-tight) were used. (C) For HEYL a vector for transposon mediated insertion was used containing Flag-Strep-tagged HEYL under control of a tetracycline responsive promoter. (D) For HEY1 a lentiviral vector containing Flag-TEV-HA tagged HEY1 was used in some experiments with identical results. (E) HEY protein expression was verified by Western Blot and real-time RT-PCR of stable cell lines. Cells were harvested after induction with different doxycycline concentrations for 48 h using standard SDS lysis buffers. Western blots on nitrocellulose membranes were developed using the Flag-M2 antibody (Sigma-Aldrich) and anti-mouse-POD as a secondary antibody (Chemicon, Millipore, Billerica, MA, USA) with chemiluminescent detection. The fold induction compared to endogenous HEY mRNA levels is shown for the concentrations used for ChIP and RT-PCR experiments (the primers used here amplify endogenous as well as transgene derived HEY transcripts). The high induction seen for HEY2 and HEYL is due to the rather low endogenous expression. (TIF) [file pgen.1002728.s001.tif]

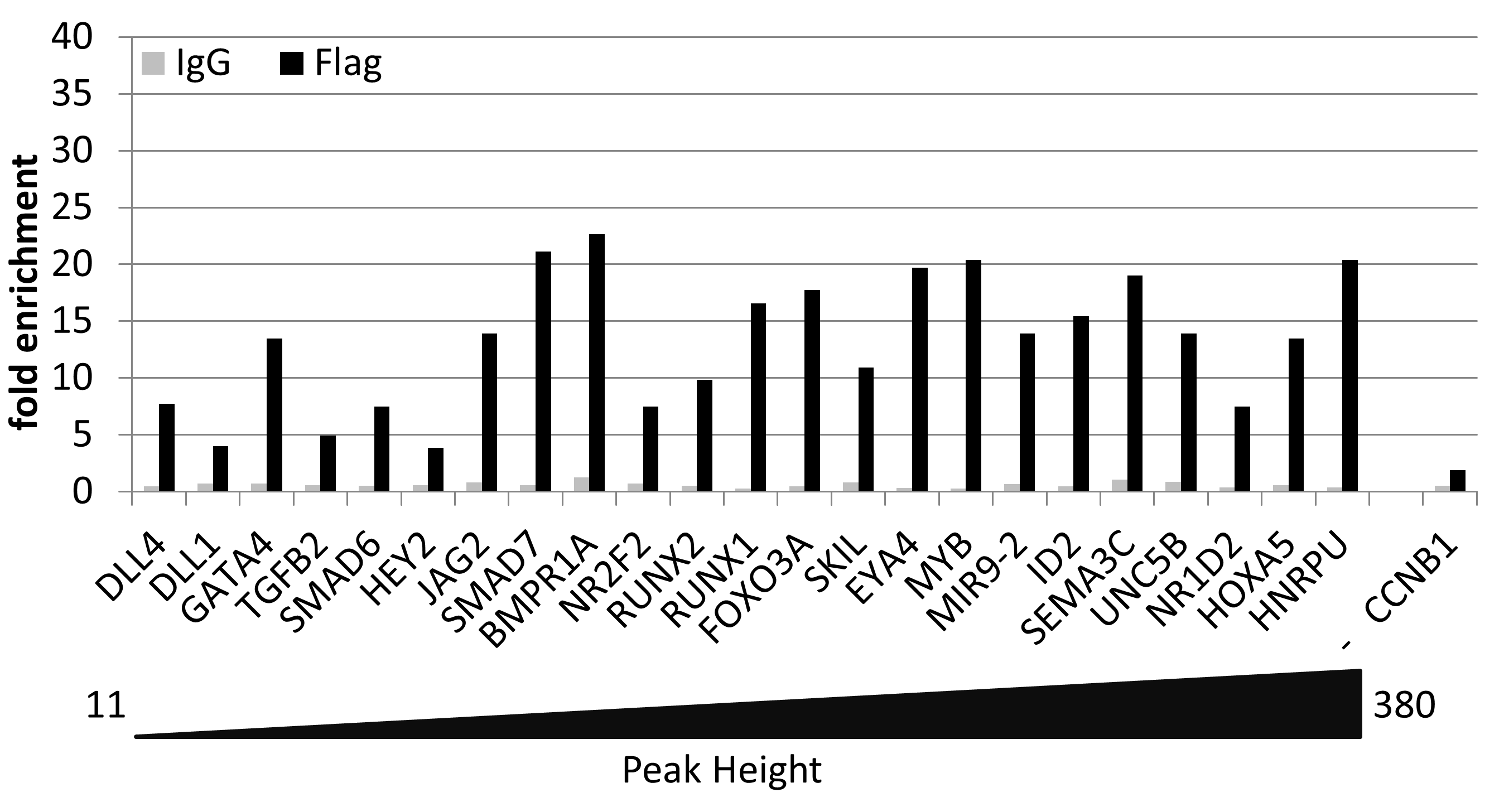

Supplement: Figure S2 — Validation of HEY1 ChIPseq data by quantitative PCR. Shown is the fold enrichment of potential HEY1 binding sites from promoter regions identified by ChIPseq compared to non-induced control cells. Genes are ordered according to peak height (from ChIPseq data) as indicated below. CCNB1 was used as a negative control. (TIF) [file pgen.1002728.s002.tif]
